# Layered Double Hydroxide Nanoplatelets with Excellent Tribological Properties under High Contact Pressure as Water-Based Lubricant Additives

**DOI:** 10.1038/srep22748

**Published:** 2016-03-08

**Authors:** Hongdong Wang, Yuhong Liu, Zhe Chen, Bibo Wu, Sailong Xu, Jianbin Luo

**Affiliations:** 1 State Key Laboratory of Tribology, Tsinghua University, Beijing 100084, China; 2 State Key Laboratory of Chemical Resource Engineering, Beijing University of Chemical Technology, Beijing 100029, China

## Abstract

High efficient and sustainable utilization of water-based lubricant is essential for saving energy. In this paper, a kind of layered double hydroxide (LDH) nanoplatelets is synthesized and well dispersed in water due to the surface modification with oleylamine. The excellent tribological properties of the oleylamine-modified Ni-Al LDH (NiAl-LDH/OAm) nanoplatelets as water-based lubricant additives are evaluated by the tribological tests in an aqueous environment. The modified LDH nanoplatelets are found to not only reduce the friction but also enhance the wear resistance, compared with the water-based cutting fluid and lubricants containing other particle additives. By adding 0.5 wt% LDH nanoplatelets, under 1.5 GPa initial contact pressure, the friction coefficient, scar diameter, depth and width of the wear track dramatically decrease by 83.1%, 43.2%, 88.5% and 59.5%, respectively. It is considered that the sufficiently small size and the excellent dispersion of NiAl-LDH/OAm nanoplatelets in water are the key factors, so as to make them enter the contact area, form a lubricating film and prevent direct collision of asperity peaks. Our investigations demonstrate that the LDH nanoplatelet as a water-based lubricant additive has a great potential value in industrial application.

Environment-friendly and economic lubricants have attracted great concerns in the field of tribology^1^,^2^,^3^ Although water is an abundant resource in our living environment with many superiorities such as high thermal conductivity, non-inflammability and easy cleaning, its extremely low viscosity and pressure-viscosity make the lubricating film 1/100–1/1000 thick of any other known hydrodynamic lubricating film in the same condition^4^,^5^ Especially under high contact pressure, effective lubricating film can be hardly formed. Furthermore, the corrosive property of water is also a critical issue in industrial production. Given that nanoparticles have been widely investigated in the field of tribology and exhibited their unique tribological properties^6^,^7^,^8^,^9^,^10^, it is reasonable to consider dispersing appropriate nanoparticles in water to improve tribological properties. In the past few years, a wide variety of layered nanoparticles, including MoS_2_ 11,12 , WS_2_^13^,^14^ and multilayer graphene^15^, were reported as extreme pressure or antifriction lubricant additives owing to their layered structure and relatively weak van der Waals force between layers. In addition, when mineral powders such as serpentine^16^,^17^ and attapulgite^18^ were added to liquid lubricants, they exhibited excellent tribological properties because an extremely hard and lubricious oxide layer was formed on the worn surface. Since layered double hydroxides (LDHs) are a big family of attractive layered clays, and have similar chemical composition and crystal structure with serpentine and attapulgite, it is of great interest to utilize the LDH platelet as a new lubricant additive. LDHs have the general formula of [M^2+^_1−*x*_M^3+^_*x*_(OH)_2_]^*x*+^(A^n−^)_*x*/n_· *m*H_2_O, where M^2+^and M^3+^are divalent and trivalent metal cations, respectively, and A^n−^ is the charge-balance anion located in the interlaminar region. The unique diversity of LDHs in varying metal cations, molar ratio and interlayer anion, has endowed the LDH-based materials with extensive applications including catalysts, ion exchange hosts, fire retardant additives, polymer/LDH composites^19^,^20^ However, the LDH particle as a water-based lubricant additive has not been reported. Furthermore, it is the fact that most of existing additives were limited in the use as oil-based lubricant additives because they would precipitate or aggregate in an aqueous environment for their relatively large size and innately hydrophobic property. Therefore, it is indeed challenging to obtain a stable aqueous suspension of nanoparticles and prevent collision of asperities in the regime of boundary lubrication.

Aiming at this challenge, here we first report a microemulsion method to synthesize LDH nanoplatelets as lubricant additives in water. The structure and chemical property of nanoparticles are analysed with X-ray diffraction (XRD), inductively coupled plasma optical emission spectroscopy (ICP-OES), Fourier transform infrared spectroscopy (FT-IR), thermogravimetric analysis (TGA), transmission electron microscopy (TEM), and technique of zeta potential. A Si_3_N_4_ ball and an Al_2_O_3_ disk are selected for tribological evaluation to avoid the corrosivity of water. The contact region after each test is analysed with white light interfering profilometer, scanning electron microscope (SEM), optical microscope and X-ray Photoelectron Spectroscopy (XPS). Based on the above results from friction tests and surface analysis, a lubrication model of LDH nanoplatelets is proposed at last.

## Results

### The structure and chemical property of LDH nanoplatelets

**Figure 1:**
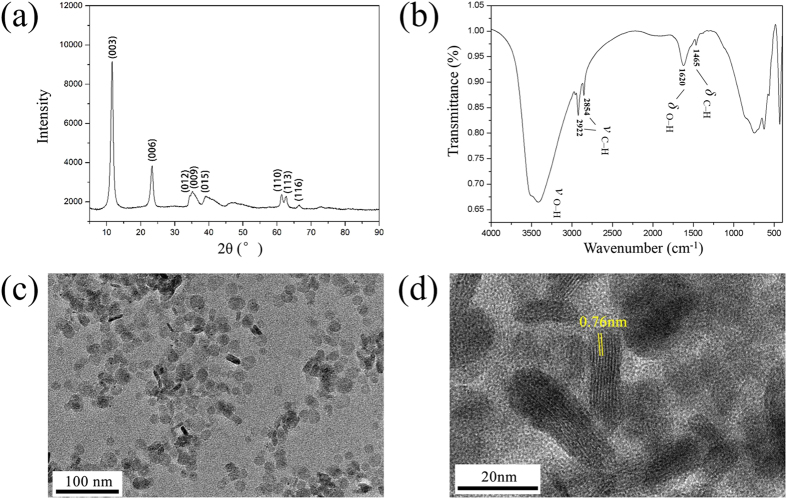
The characterizations of the as-synthesized NiAl-LDH. (**a**) The XRD pattern of the as-synthesized NiAl-LDH with peak (003) at 11.6 ° and peak (110) at 61.4 °. (**b**) The FT-IR spectroscopy of the as-synthesized NiAl-LDH with *ν*_O–H_ around 3400 cm^−1^, *δ*_O–H_ at 1620 cm^−1^, *δ*_C–H_ at 1465 cm^−1^ and *ν*_C–H_ at 2854 and 2922 cm^−1^. (**c**) The TEM imagine of the as-synthesized NiAl-LDH, which contains standing part and horizontal part. (**d**) The high-resolution TEM image shows the layered structure and the vertical size of nanoplatelets.

The NiAl-LDH/OAm nanoplatelets were synthesized by a microemulsion method^21^ (see Methods for details). The XRD pattern shown in Fig. 1a exhibits a series of typical reflection peaks of rhombohedral phase, confirming the structure of NiAl-LDH. A hexagonal unit cell *a = b* = 2*d*_(110)_ = 0.302 nm and the basal interlayer distance (003) of 0.762 nm (see Supplementary Table S1) strongly indicate the intercalation of Cl^−^ anions in the interlayer galleries^22^ Chemical formula is identified as [Ni_1.97_Al_1.04_(OH)_6_]Cl_1.04_· 0.5 H_2_O· (oleylamine)_0.078_ by analysing the mass fraction of main elements (see Supplementary Table S2). FT-IR spectroscopy was applied to evaluate the surface modification of the NiAl-LDH sample. Figure 1b shows that the stretching vibration (*ν*_O–H_) around 3400 cm^−1^ and the bending vibration (*δ*_O–H_) at 1620 cm^−1^ are visible, which can be ascribed to the water molecules in the LDH interlayer galleries and the hydroxide groups of the layer surface. These bands might overlap with the infrared vibrational peaks at 3300 cm^−1^ (*ν*_N–H_) and 1593 cm^−1^ (*δ*_N–H_) for the oleylamine molecule, but the bending vibration of methyl group at 1465 cm^−1^ (*δ*_C–H_) and the hydrocarbon stretching vibration at 2854 and 2922 cm^−1^ (*ν*_C−H_) correspond exactly with the characteristic peaks of oleylamine^23^ Considering that the oleylamine molecule is the only material containing nitrogen element, as detected in Supplementary Table S2, we therefore think that the surface of as-synthesized sample is successfully modified by oleylamine molecules. To assess the thermal stability of LDH nanoplatelets, thermogravimetric analysis (TGA) was performed and the result shows that weight loss mainly occurs in two regions (see Supplementary 3). In the first region (30–189 °C), the weight loss can be attributed to some adsorbed water and interlayer water. In the second region (189–800 °C), the weight loss is mainly caused by the decomposition of OH^−^ in the layer^24^ Because the particles are applied in an aqueous environment and water has a high specific heat capacity, the relatively low operating temperature will not have an influence on the thermal stability of nanoplatelets.

### Particle size and dispersion effect

**Figure 2:**
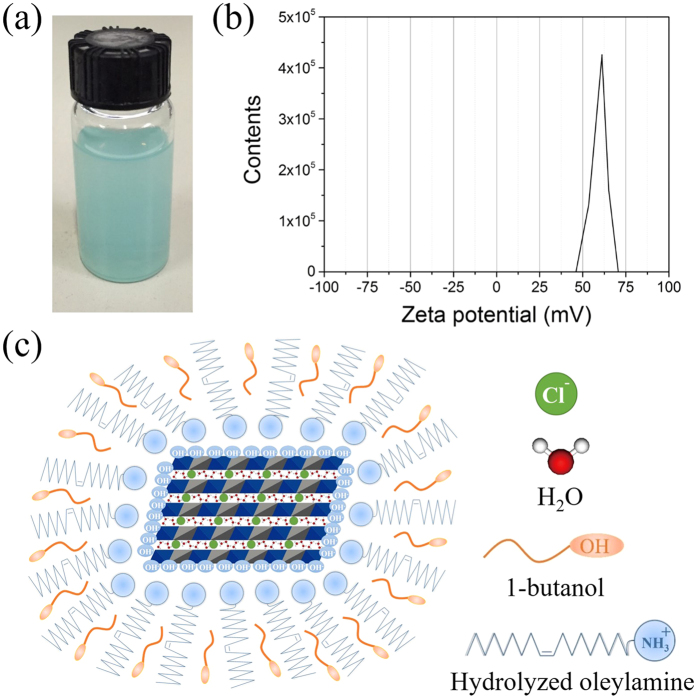
The dispersion effect and speculative model of NiAl-LDH/OAm nanoplatelets in water. (**a**) A translucent solution with NiAl-LDH nanoplatelets dispersed in water. (**b**) Zeta potential of the as-synthesized NiAl-LDH dispersed in water. (**c**) Schematic model of NiAl-LDH/OAm. The nanoplatelets are stabilized *via* electrostatic interaction between the polarized OH^−^ groups of NiAl-LDH and the -NH_3_^+^head groups of oleylamine. -OH groups of assistant surfactant 1-butanol reduce the hydrophobicity of nanoplatelets.

When the as-synthesized particles were dispersed in water without any extra dispersion or surfactant agents, a translucent solution was obtained with no precipitate (see Fig. 2a). In order to assess the stability of dispersion, we measured the zeta potential of the suspension containing 0.5 wt% NiAl-LDH/OAm nanoplatelets. The zeta potential is determined to be +59.6 mV (see Fig. 2b). This value is far beyond the range from −40 to +40 mV, which is the typical feature of a stable suspension or colloid solution^25^. In addition, when mass fraction is no more than 1.0 wt%, the obtained pH 7.2–7.3 of aqueous solution is suitable for LDHs to stabilize. On account of good dispersibility in water, the TEM image (see Fig. 1c) shows a uniform distribution of typical platelet-like samples, which contain standing and horizontal morphology. Meanwhile, the high-resolution TEM (HRTEM) image clearly depicts the layered structure and the vertical size of NiAl-LDH nanoplatelets (see Fig. 1d). According to the statistical results by measuring fifty different samples (see Supplementary 4), the average size of nanoplatelets is *ca.* 19.42 nm wide and *ca.* 8.59 nm thick by Gaussian distribution fitting. The good dispersion effect of nanosized Ni-Al LDH are closely related to the microemulsion method we use. In this synthesis, oleylamine plays a role in the following parts^21^: (i) providing alkaline environment for the precipitation of LDH *via* the protonation of amine; (ii) forming reverse micelles in the water/oil mixture for crystal nucleation and growth; (iii) excess oleylamine can be recycled for continued use. We believe that the protonated amine head groups are oriented together to form nanosized reactors, because the polarized OH^−^ groups of NiAl-LDH and the -NH_3_^+^ head groups of oleylamine are linked *via* electrostatic interaction^26^ As an assistant surfactant, 1-butanol also plays an essential role in the reaction. The hydrophobic head groups of 1-butanol molecule are oriented towards the peripheral alkyl end groups of the reverse micelles, and intercalated among the long-chain alkyl tails of oleylamine *via* Van der Waals interaction. Thus, it can reduce the repulsion between -NH_3_^+^ head groups of the oleylamine, and -OH groups of 1-butanol will slightly lower the hydrophobicity of reverse micelles. On the basis of the above results, the speculative model of NiAl-LDH/OAm nanoplatelets in water is illustrated in Fig. 2c.

### Tribological properties of NiAl-LDH

**Figure 3:**
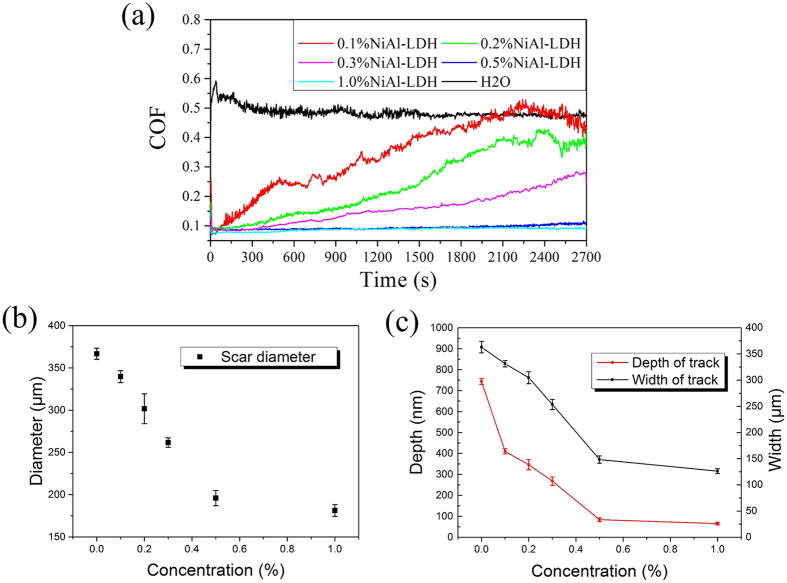
The results of the tribological tests of NiAl-LDH aqueous solutions with different weight fraction from 0.1 wt% to 1.0 wt%. (**a**) Friction coefficient of NiAl-LDH aqueous solutions with different weight fractions. (**b**) The wear scar diameter of Si_3_N_4_ balls after tests. (**c**) The depth and width of Al_2_O_3_ disk wear tracks after tests. The load is 2N, and the average speed is 0.024 m/s.

To investigate the tribological properties of Ni-Al LDH/OAm nanoplatelets as water-based lubricant additives under high contact pressure, the friction coefficient (COF) between ceramic surfaces, lubricated by different mass fractions of NiAl-LDH aqueous solutions from 0.1 wt% to 1.0 wt% under 1.5 GPa initial contact pressure, is displayed in Fig. 3a. Given the low concentration of nanoparticles in water, the bulk viscosity of fluids (0.897 cP at 25 °C) makes no difference with that of pure water, which suggests that the lubrication stage is in the regime of boundary lubrication throughout the experiment. The diameter of wear scar (see Fig. 3b) and the size of wear track (see Fig. 3c) after each friction test are measured to evaluate the antiwear property. From these charts, certain aqueous suspensions exhibit excellent tribological performance, while the lower concentration leads to a rising trend of COF. It can be deduced that the increasing concentration of particles improves the tribological performance. When the weight fraction of NiAl-LDH nanoplatelets is no less than 0.5 wt%, the COF of its aqueous solution will keep relatively low and stable. For pure water, the mean friction coefficient reaches 0.576 in 45 minutes. Meanwhile, the wear scar diameter of Si_3_N_4_ ball is 0.361 mm; the track is 0.748  μm deep and 0.363 mm wide. In sharp contrast, the mean friction coefficient of 0.5 wt% NiAl-LDH/OAm aqueous solution is 0.0974, which reduces by 83.1%. Moreover, the scar diameter (0.205 mm) reduces by 43.2%; the depth (0.086 μm) and width (0.147 mm) of the track decrease by 88.5% and 59.5%, respectively. In addition, the relationship between friction coefficient and average linear speed was analysed (see Supplementary 5). It shows that the NiAl-LDH nanoplatelet as lubricant additive still works at low speed, and the COF of its 0.5 wt% aqueous solution can keep around 0.1 when the average linear speed is higher than 0.012 m/s. As a result, NiAl-LDH nanoplatelets take a significant effect on improving both friction-reducing and antiwear performance.

**Figure 4:**
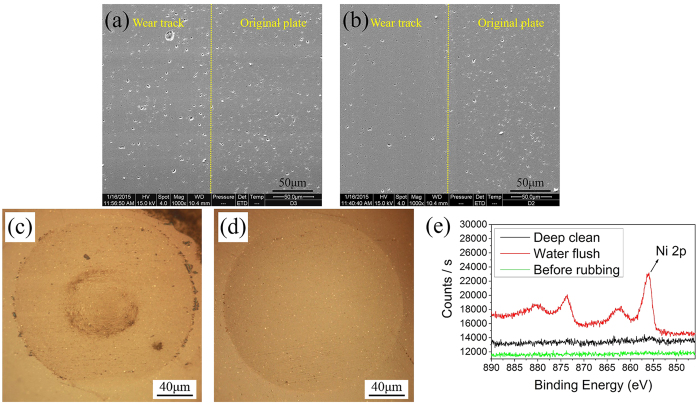
The details of sliding surfaces and surface analysis. The SEM images of the border of wear track on the disk tested by (**a**) pure water; (**b**) 0.5 wt% NiAl-LDH aqueous solution. The optical images of wear scar on the ball (**c**) after water flush; (**d**) after deep clean. The load is 2N and the average speed is 0.024 m/s. (**e**) XPS spectrum of the ball before tribological experiment, the wear scar after water flush and the wear scar after deep clean, respectively. It shows that the tribofilm is not formed, and no obvious chemical reaction occurs during the friction test.

After friction tests, the wear track was cleaned by ultrasonic water, and it was observed by scanning electron microscope. The SEM images provided in Fig. 4a,b show the microstructure of rubbing surfaces and borders. As compared with the initial surface of disk, there are more random micro-pits in the ceramic disk lubricated by pure water. However, lubricated by the NiAl-LDH aqueous solution, the surface get smoother, and fewer defects are found in the track. The disappeared defects in the track indicate that the direct collision of asperities is prevented in the process of friction. With the aim to assess the role of NiAl-LDH nanoplatelets in lubricating stage, the wear scar after tests were washed by flowing water and observed by an optical microscope (see Fig. 4c). It is found that some particles adhere to the rubbing surface. However, the wear scar turns clear (see Fig. 4d) after it is deep cleaned by ultrasonic water and ethanol separately for 5 minutes. According to the XPS results of this region before and after friction test (see Fig. 4e), the element of Ni can only be detected in the wear scar after water flush. After the wear scar was deep cleaned, the disappeared peak of Ni2p indicates that the tribofilm is not formed. Therefore, it can be considered that NiAl-LDHs are physically adsorbed on the sliding surface. Then, these particles were collected and analysed (see Supplementary 6). The results of the TEM imagine and electron diffraction pattern demonstrate its original structure after friction test. Thus, it can be deduced that no obvious chemical reaction occurs throughout the tribological experiment.

**Figure 5:**
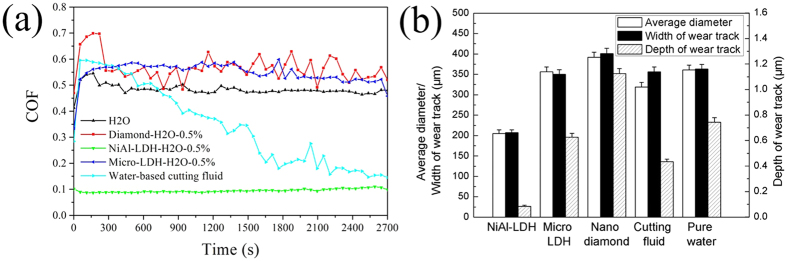
The results of the tribological tests of five lubricant samples, which contain the diamond nanoparticles, NiAl-LDH nanoplatelets, LDH microplatelets, water-based cutting fluid and pure water. The weight fraction of particle additives in each lubricant is 0.5 wt%. The load is 2N, and the average speed is 0.024 m/s. (**a**) Friction coefficient of five lubricant samples. (**b**) The wear scar diameter of Si_3_N_4_ balls, the width and depth of wear tracks with different lubricants after tests. The error bar in (**b**) corresponds to a standard deviation in the measurement.

In order to make comparison with other lubricating fluids, aqueous solution of 0.5 wt% diamond nanoparticles^27^ (TEM image provided in Supplementary Fig. S7a), 0.5 wt% LDH microplatelets (SEM image shown in Supplementary Fig. S7b) and water-based cutting fluid containing 20 wt% polyether, which is commonly used in industrial production^2^,^28^, are tested in the same condition, and their COFs are recorded in Fig. 5a. After friction experiments, the wear scar diameter of Si_3_N_4_ balls and the size of wear tracks are collected in Fig. 5b. It is quite interesting that the COF of cutting fluid declines gradually with time going on, while a rise of COF occurs after diamond nanoparticles and LDH microplatelets are added in water. Since the worn surface lubricated by water-based cutting fluid becomes a *ca.* 319 μm diameter flat, the load pressure turns to about 25 MPa at last. Through calculation (see Supplementary 8), the boundary lubrication of cutting fluid at initial stage has transformed to the stage of mixed lubrication in the end. As for LDH microplatelets, on account of their relatively large size, they almost have no chance to enter the contact area. However, unsuitable nanoparticle additives under such high pressure like diamond nanoparticles may even lead to a higher COF and worse wear condition.

## Discussion

**Figure 6:**
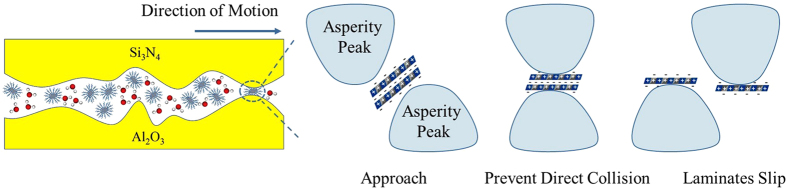
Schematic illustration of the lubrication model. In friction period, the nanoplatelets between asperity peaks prevent surface from direct collision. Exfoliated sheets assemble on the sliding surfaces, and the lubricating film is formed in contact area.

In previous studies^29^,^30^,^31^, several reports show that the coefficient of friction of self-mated ceramics under aqueous condition could decrease from a relatively high value to an ultra-low range (<0.03) after a certain running-in process, where tribochemical reaction occurs. The formation of a thin film between sliding surfaces leads to the initiation of hydrodynamic lubrication. The smooth surface, high speed and extremely low contact pressure (several MPa) are indispensable conditions for the low friction. However, it is quite different with our condition, where the lubrication stage always maintains in the regime of boundary lubrication, and its applicable contact pressure can reach 1.5 GPa, far higher than the above mentioned value, with no need for running-in process. Thus, it is necessary to investigate the lubrication mechanism of our system. With this purpose, the aqueous solution after friction test was collected, and particles are analysed in Supplementary 9. It proves that large quantities of LDH nanoplatelets are exfoliated and the sheets are composed of limited layers (n < 5)^32^. Based on the analysis of LDH composition, the electrostatic force among the interlayer galleries is relatively weak for the existence of water molecules^33^, so laminates are likely to slip under strong shear force. However, laminates still remain intact because of their strong covalent bonds. As illustrated in Fig. 6, when asperity peaks meet and separate, generating strong shear force, the nanoplatelets will be exfoliated and sheets will assemble on the rubbing surface due to their large specific surface area, unsaturated bonds and dangling bonds so as to prevent direct contact of asperity peaks. Given the XPS analysis in the wear scar, it is believed that a lubricating film is formed by nanoplatelets on the sliding surfaces, and it physically prevents the collision of asperities under boundary lubrication. In summary, the excellent tribological properties of NiAl-LDH additives under high pressure in water-based system are attributed to the following points: (1) sufficiently small dimensional size for entering the contact area and forming a lubricating layer between asperities, (2) excellent dispersion to ensure the continuous providing of nanoplatelets during the reciprocating motion, (3) both layer structure and high specific surface area of nanoplatelets to absorb on the rubbing surface so as to prevent direct collision of asperities, (4) laminates strong enough to carry load. Therefore, the NiAl-LDH nanoplatelet is expected to be a beneficial lubricant additive to undertake load, reduce friction and resist abrasion.

In this paper, NiAl-LDH nanoplatelets, which have few ordered layers with its lateral size of *ca.* 19.42 nm, were synthesized in a microemulsion by a hydrothermal method and surface modified successfully so that we obtained a stable and translucent solution without additional dispersion or surfactant agents. Because of small size and excellent dispersion, a good lubricating layer forms in contact area under 1.5 GPa contact pressure. In comparison with pure water, the friction coefficient, wear scar diameter, depth and width of wear track decrease by 83.1%, 43.2%, 88.5% and 59.5%, respectively. Thus, we can draw the conclusion that both friction-reducing and antiwear property under high contact pressure can be greatly improved by NiAl-LDH nanoplatelets. This work enriches the research about water-based lubricants and has potential value in energy saving, machining, equipment operation and other industrial applications.

## Methods

### Synthesis

NiAl-LDH nanoplatelets were synthesized by a microemulsion method. Deionized water was used in the whole experiment. The procedure is as follows: A 15 ml mixed saline solution of NiCl_2_·6H_2_O and AlCl_3_·6H_2_O ([Ni^2+^] = 1.5 M, [Al^3+^] = 0.75 M) was added dropwise to a stirred mixed solution of oleylamine (15 mL; Sigma-Aldrich) and 1-butanol (15 ml) under the protection of N_2_ atmosphere. After the mixture was stirred for 15 min, transferred into an autoclave and heated at 120 °C for 24 hours. The precipitation was collected by centrifugation, washed three times with a mixture of ethanol and distilled water (1:1), and dried at 80 °C overnight.

### Characterization of LDH Nanoplatelets

The powder X-ray diffraction (XRD) pattern was recorded with a Bruker D8 Advance diffractometer in reflection mode (Cu Kα radiation, λ = 1.54 Å) over a 2θ range of 3–70 °C. Metal element analysis was carried out using inductively coupled plasma optical emission spectroscopy (ICP-OES). Contents of C, H and N elements were measured using a Vario EL III in combustion mode in the range of 950–1200 °C. FT-IR spectroscopy was recorded on a Nicolet 6700 FT-IR spectrometer (4000–400 cm^−1^) using the KBr pellet technique. Thermogravimetric analysis (TGA) was performed by the TGA/DSC1, STAR^e^ system (Mettler Toledo) under a nitrogen atmosphere from 30 to 800 °C with a heating rate of 10 °C min^−1^. Zeta potential of NiAl-LDH aqueous solution (0.5 wt%) was measured by Malven Zetasizer Nano ZSI instrument at 25 °C. TEM was observed on JEM 2010 with an accelerating voltage of 120 kV. Particles were dispersed in water with ultrasonic and spread on carbon film, then dried at 80 °C for 10 hours in air. The bulk viscosity of solution was measured with a standard rheometer (MCR301, Anton Paar Physica).

### Tribological Experiments and Characterization

Universal Micro-Tribotester (UMT-3, CETR) in a reciprocating ball-on-disk mode recorded friction coefficient every 0.04 s. The load of ball was 2N, corresponding to a maximum Hertzian contact pressure of 1.512 GPa (far more than the commonly practical pressure in industry). The reciprocation of the disk was controlled by a motor with the average linear speed of 0.024 m/s, as the frequency was 4 Hz while the stroke of reciprocation was 3 mm. The load and friction force in the process of sliding were recorded accurately by a sensor with the precision of 2.5 mN. The ball was made of Si_3_N_4_ with diameter of 4 mm, and surface roughness (Sq) of 16.5 nm. The disk was made of Al_2_O_3_ ceramic with surface roughness (Sq) of 10.9 nm. The ball and the disk were ultrasonically cleaned with water and ethanol separately for 10 minutes. The contact region between ball and disk during the test was fully submerged in liquids. Each test lasted 45 minutes and the temperature was controlled at 25 °C in the whole process. To ensure repeatability, all experiments were conducted at least three times. After tribological tests, the sliding surface was washed by deionized water for 10 minutes and dried. The morphology of contact region was observed by white light interfering profilometer (MICROXAM-3D) and a scanning electron microscope (SEM, QUANTA 200 FEG). An optical microscope (Olympus BX60) was used to observe the wear scar of ball. The particles adhering to the surface of wear scar were characterized by X-ray Photoelectron Spectroscopy (XPS). The XPS spectra were obtained by an ESCALAB 250 XI (Thermo Scientific Instrument) with a monochromatic aluminium X-ray source. 1s peak of C at 285 eV was used to calibrate all binding energy values. The acceleration voltage of argon ion beam and current of it were 1000 eV and 10 μA, respectively.

## Additional Information

**How to cite this article**: Wang, H. *et al*. Layered Double Hydroxide Nanoplatelets with Excellent Tribological Properties under High Contact Pressure as Water-based Lubricant Additives. *Sci. Rep.* **6**, 22748; doi: 10.1038/srep22748 (2016).

## Supplementary Material

Supplementary Information
