# Supplementary material for: Layered Double Hydroxide Nanoplatelets with Excellent Tribological Properties under High Contact Pressure as Water-Based Lubricant Additives
Source: Sci Rep. 2016 Mar 8;6:22748. doi: 10.1038/srep22748 (PMC4782130; doi:10.1038/srep22748)
Supplement: Supplementary Information [file srep22748-s1.pdf]

Supplementary Information

Layered Double Hydroxide Nanoplatelets with Excellent Tribological Properties under High Contact Pressure as Water-based Lubricant Additives

Hongdong Wang, Yuhong Liu, Zhe Chen, Bibo Wu, Sailong Xu, Jianbin Luo

1. Lattice parameters of NiAl-LDH.

Lattice parameters of NiAl-LDH are shown in Supplementary Table S1.The position of the (110) Bragg reflection shows the feature of layer structure with a hexagonal unit cell $a=b=2d_{(110)}=0.302$ nm. The basal interlayer distance (003) is calculated to be 0.762 nm, strongly indicating the intercalation of Cl^- anions between the interlayer galleries. [S1]

NiAl-LDH	$d_{(003)}$	$d_{(006)}$	$d_{(009)}$	$d_{(110)}$	a	c
(nm)	0.762	0.382	0.256	0.151	0.302	2.286

Table S1. Lattice parameters of NiAl-LDH.

2. Elemental analysis for NiAl-LDH.

Supplementary Table S2 lists the mass fraction of main elements. Metal element analysis was carried out using inductively coupled plasma optical emission

spectroscopy (ICP-OES).Contents of C and H elements were measured using a Vario EL III in combustion mode in the range of 950–1200 ℃.

Element	Ni	Al	C	H	N
(wt%)	35.00	8.58	5.43	3.04	0.38

Table S2. Elemental data for NiAl-LDH

3. The thermogravimetric analysis (TGA) of NiAl-LDH/OAm nanoplatelets.

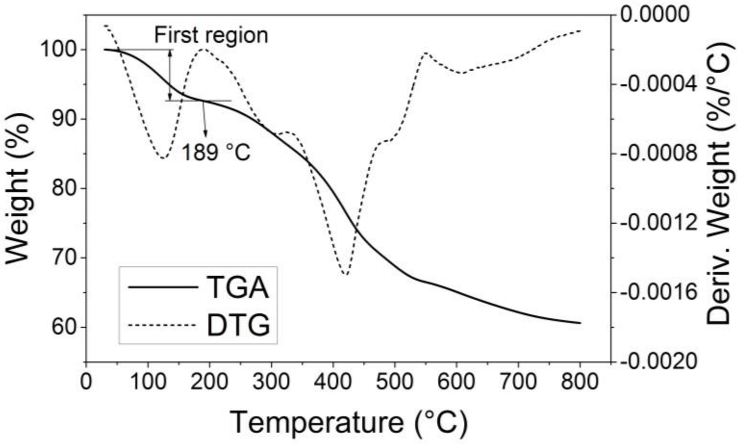

Figure S3. The TGA-DTG curve of the as-synthesized NiAl-LDH from 30 to 800 ℃.

The thermogravimetric analysis (TGA) was performed to assess the thermal stability of LDH nanoplatelets. In the TGA-DTG curve, two main regions of weight loss can be clearly observed. The weight loss in the first region (30–189 ℃) can be attributed to some adsorbed water and interlayer water. The weight loss in the second region (189–800 ℃) is mainly caused by the decomposition of OH^- in the layer. [S2] Therefore, the nanoplatelets in an aqueous environment will be thermally stable throughout the experiment.

4. The size of as-synthesized NiAl-LDH.

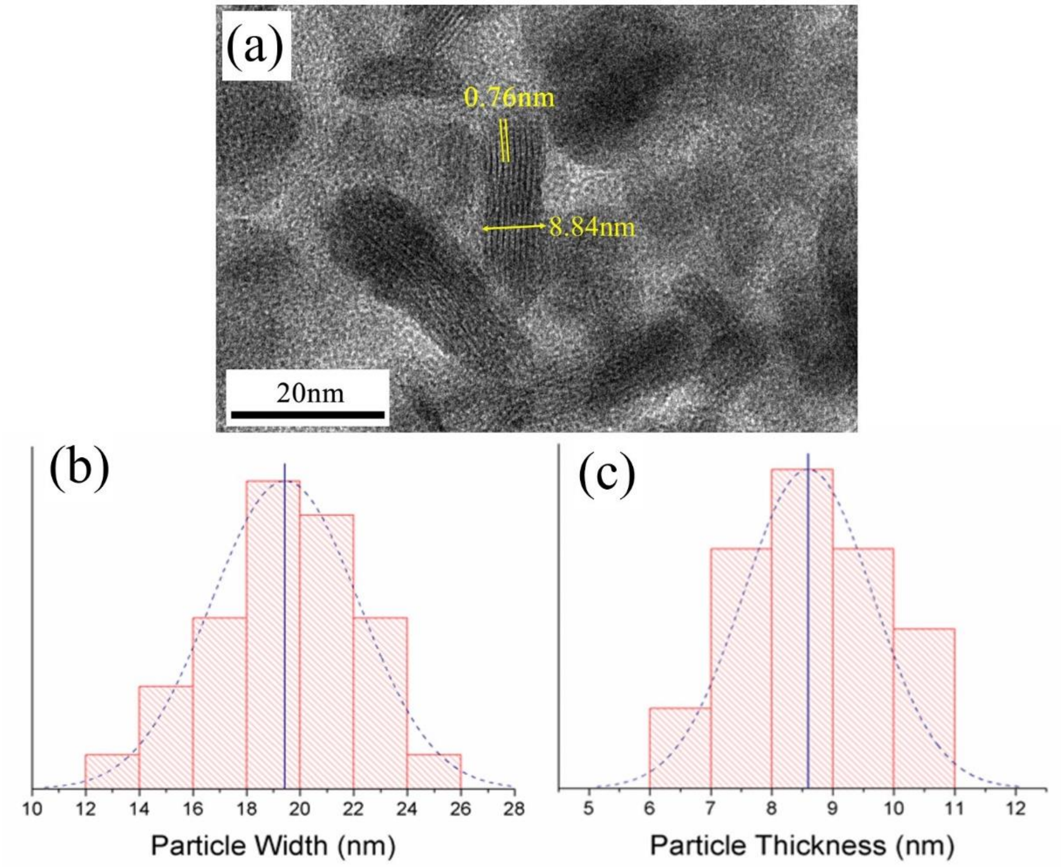

Figure S4. (a) The details of NiAl-LDH in TEM imagines. Distribution statistics of (b) particle width; (c) particle thickness.

Details of the NiAl-LDH nanoplatelets displayed in Supplementary Fig. S4a further reveals the layered structure, which are composed of several well ordered nanosheets stacking together. The interlayer distance is determined to be 0.76 nm, well consistent with the result of the XRD pattern. The average width and thickness of nanoplatelets, by measuring fifty different NiAl-LDH nanoplatelets (see Supplementary Fig. S4b and Fig. S4c), are determined to be 19.42 nm (σ=3.01 nm) and 8.59 nm (σ=1.06 nm), respectively.

5. The relationship between friction coefficient and average linear speed.

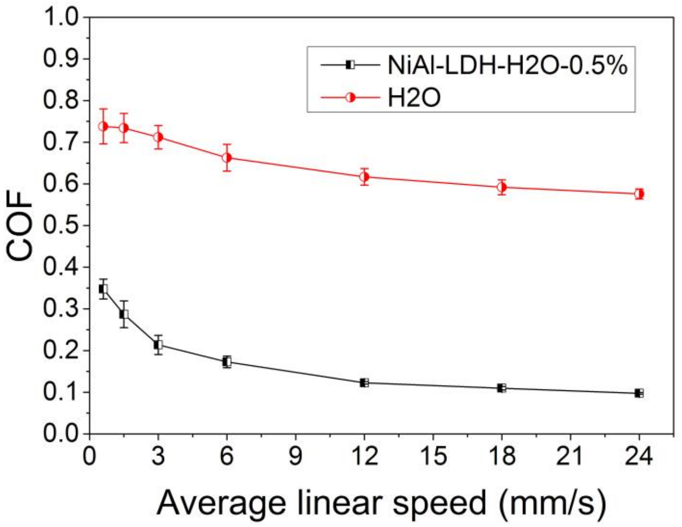

Figure S5. The friction coefficient of pure water and 0.5 wt% NiAl-LDH/OAm aqueous solution under different average linear speeds. The error bar corresponds to a standard deviation in repetitive results.

The average linear speed is controlled by changing the frequency of reciprocation. The friction coefficient of pure water and 0.5 wt% NiAl-LDH/OAm aqueous solution under different average linear speeds is displayed in Fig. S5. It is clearly shown that the friction coefficients will have a rise during deceleration, and the NiAl-LDH nanoplatelet as lubricant additive still improves the tribological property at low speeds. The friction coefficient of 0.5 wt% NiAl-LDH/OAm aqueous solution can keep around 0.1 when the average linear speed is higher than 12 mm/s.

6. The morphology and structure of the particles on the sliding surface after friction test.

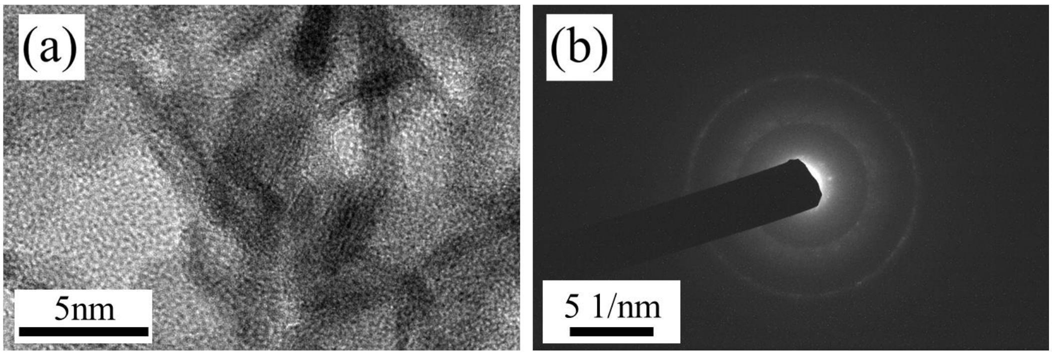

Figure S6. (a) The TEM imagine of the particles on the sliding surface after friction test. (b) The electron diffraction pattern of particles.

The particles absorbing on the sliding surface after friction test were observed by the high-resolution transmission electron microscopy. The electron diffraction pattern was obtained to determine whether the structure of them made a difference. Although the layered structure of agglomerated particles are difficult to distinguish through the TEM image, the electron diffraction pattern shows that the crystal structure of them correspond with the original results of XRD (d_{110}, d_{009}). Thus, it can be deduced that the crystal structure of particles keeps stable during rubbing process.

7. The morphology of diamond nanoparticles and LDH microplatelets.

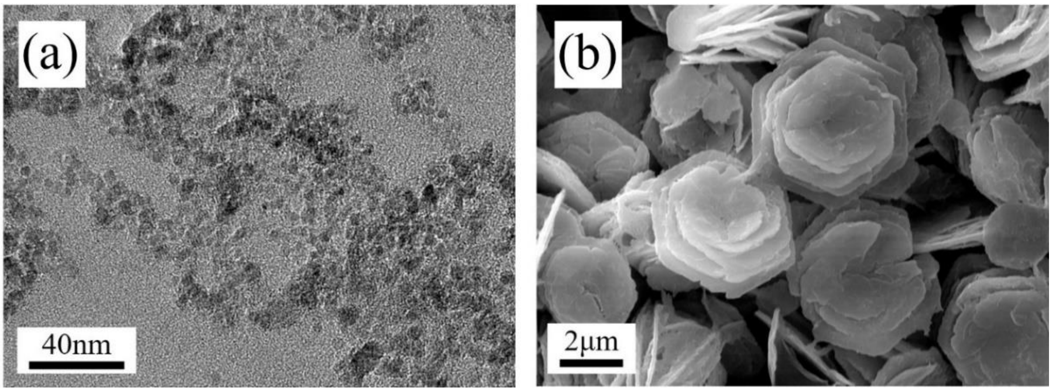

Figure S7. (a) The TEM imagine of diamond nanoparticles. (b) The SEM image of LDH microplatelets.

The diamond nanoparticles, whose TEM image is supplied in Fig. S7a, were obtained by explosive detonation. Through surface hydroxylation, the diamond nanoparticles take a good dispersion effect in water. The LDH microplatelets shown in Fig. S7b were synthesized by a co-precipitation method. The lateral size of platelets is *ca.* 3 μm and layer structure can be observed clearly.

8. The lubricating stage of water-based cutting fluid.

At the start of the test, the minimun film thickness can be calculated on the basis of Hamrock−Dowson theory by the formula[S3]

$$h_{\min}=3.63\frac{U^{0.68}G^{0.49}R}{W^{0.073}}(1\text{-}e^{-0.68k}) \qquad (1)$$

where $U=\eta V/E'R$, $G=\alpha E'$, $W=F/E'R^2$, R (=2 mm) is the radius of the ball, V (≈24 mm/s) is the relative velocity of sliding surface, η (≈5.62 cP at 25 ℃) is the bulk viscosity of fluid, α (≈2.5 GPa^{-1} [S4,S5]) is the viscosity-pressure coefficient, E' is the effective modulus of elasticity, F is the normal load, and k (≈1) is the ellipticity. At final stage, because of the wear scar generated after friction test, the minimun film thickness can not be obtained directly by formula 1. However, here we consider the wear scar as the Hertz elastic deformation of a larger ball under the same normal force. [S6] The radius of the equivalent ball (R') can be calculated as

$$R' = (a'/a)^3 R \qquad (2)$$

where a (≈0.025 mm) is the radius of Hertz contact area, a' (≈0.16 mm) is the ridius of the wear scar. The lubrication regime can be determined by using the ratio of theoretical minimum film thickness to the combined surface roughness, which can be

expressed by the formula[S5,S7]

$$\lambda = \frac{h_{min}}{\sigma} = \frac{h_{min}}{(\sigma_1^2+\sigma_2^2)^{0.5}} \qquad (3)$$

where h_{min} is the minimum film thickness in theory, σ is the composite surface roughness; σ_1 and σ_2 are the roughness of contacting surfaces. After calculation, if the ratio $\lambda<1$, the lubrication regime is boundary lubrication; if $1<\lambda<3$, mixed lubrication; when $\lambda>3$, elastohydrodynamic lubrication (EHL) regime. [S8]

At initial stage, σ_1=16.5 nm, σ_2=10.9 nm. After calculation, the film thickness h_{min} is about 1.02 nm, deducing that λ equals 0.051. It indicates that the initial lubrication stage is boundary lubrication. However, as $\sigma_1{}'$=11.9 nm, σ_2' =6.11 nm after friction test, the estimated film thickness is 13.56 nm and the value of λ is about 1.013. Thus, the final stage is in the regime of mixed lubrication.

9. The characterization of NiAl-LDH after friction tests.

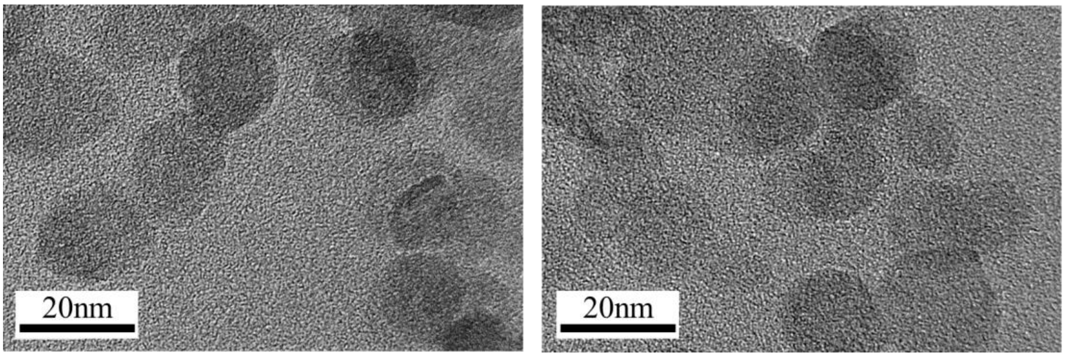

Figure S9. The TEM imagines of NiAl-LDH nanoplatelets after the friction test.

The TEM images of platelets after friction test in Fig. S9 show that they overlap with each other and the bottom particle even can be observed through the above one on

account of their ultrathin feature, which are similar with the images reported by Hu *et al.*[S9] and O'Leary *et al.*[S10], who delaminate LDHs in polar solvent under high shearing forces. It can be deduced that exfoliated sheets dispersed on the substrate are composed of limited layers ($n<5$).

References

S1. Miyata, S. Anion-Exchange Properties of Hydrotalcite-Like Compounds. *Clays Clay Miner.* **31,** 305–311 (1983).

S2. Wang, X. *et al.* New synthetic route to Mg–Al–CO_3 layered double hydroxide using magnesite. *Mater. Res. Bull.* **48,** 1228–1232 (2013).

S3. Hamrock, B. J. & Dowson, D. Isothermal Elastohydrodynamic Lubrication of Point Contacts: Part III—Fully Flooded Results. *J. Lubr. Technol.* **99,** 264 (1977).

S4. Zhang, C. H. *et al.* EHL properties of polyalkylene glycols and their aqueous solutions. *Tribol. Lett.* **45,** 379–385 (2012).

S5. Wen, S. & Huang, P. *Principles of Tribology*. (Tsinghua University Press, 2011).

S6. Chen, Z., Liu, Y., Zhang, S. & Luo, J. Controllable superlubricity of glycerol solution via environment humidity. *Langmuir* **29,** 11924–30 (2013).

S7. Dowson, D. & Jin, Z.-M. Metal-on-metal hip joint tribology. *Proc. Inst. Mech. Eng. Part H J. Eng. Med.* **220,** 107–118 (2006).

S8. Luo, J. B., Lu, X. C. & Wen, S. Z. Developments and unsolved problems in nano-lubrication. *Prog. Nat. Sci.* **11,** 173–183 (2001).

S9. Hu, G., Wang, N., O'Hare, D. & Davis, J. Synthesis of magnesium aluminium layered double hydroxides in reverse microemulsions. *J. Mater. Chem.* **17,** 2257 (2007).

S10. O'Leary, S., O'Hare, D. & Seeley, G. Delamination of layered double hydroxides in polar monomers: new LDH-acrylate nanocomposites. *Chem. Commun.* **14,** 1506–1507 (2002).
